# Long term outcomes associated with the use of perioperative systemic chemotherapy on low grade appendiceal mucinous neoplasms with pseudomyxoma peritonei treated with cytoreductive surgery and hyperthermic intraperitoneal chemotherapy

**DOI:** 10.3389/fonc.2024.1456920

**Published:** 2025-01-09

**Authors:** Samantha M. Ruff, Gyulnara Kasumova, Manoj Palavalli, Oliver S. Eng, Laura Lambert, Callisia Clarke, Sameer Patel, Jula Veerapong, Keith Fournier, Daniel Abbott, Charles Staley, Travis Grotz, Fabian Johnston, Mustafa Raoof, Sean Dineen, Jordan M. Cloyd, Alex C. Kim

**Affiliations:** ^1^ Department of Surgery, Division of Surgical Oncology, The Ohio State University Wexner Medical Center/James Comprehensive Cancer Center, Columbus, OH, United States; ^2^ Division of Surgical Oncology, Department of Surgery, Central Maine Medical Center, Lewiston, ME, United States; ^3^ Department of Surgical Oncology, University of California Irvine Medical Center, Irvine, CA, United States; ^4^ Department of Surgical Oncology, University of Utah, Salt Lake City, UT, United States; ^5^ Department of Surgical Oncology, Medical College of Wisconsin, Milwaukee, WI, United States; ^6^ Department of Surgical Oncology, University of Cincinnati, Cincinnati, OH, United States; ^7^ Department of Surgical Oncology, University of California San Diego, San Diego, CA, United States; ^8^ Department of Surgical Oncology, MD Anderson Cancer Center, Houston, TX, United States; ^9^ Department of Surgical Oncology, University of Wisconsin Medical Center, Madison, WI, United States; ^10^ Department of Surgical Oncology, Emory University Hospital, Atlanta, GA, United States; ^11^ Department of Surgery, Mayo Clinic, Rochester, MN, United States; ^12^ Department of Surgical Oncology, The Johns Hopkins University, Baltimore, MD, United States; ^13^ Department of Surgical Oncology, City of Hope Cancer Center, Duarte, CA, United States; ^14^ Department of Surgical Oncology, Moffitt Cancer Center, Tampa, FL, United States; ^15^ Department of Surgery, Division of Surgical Oncology, University of Texas Southwestern Medical Center, Dallas, TX, United States

**Keywords:** HIPEC, cytoreductive surgery, low grade appendiceal mucinous neoplasm, LAMN, chemotherapy

## Abstract

**Introduction:**

Low grade appendiceal mucinous neoplasms (LAMN) are indolent tumors that lack invasive potential but may present as pseudomyxoma peritonei. Cytoreductive surgery (CRS) and hyperthermic intraperitoneal chemotherapy (HIPEC) significantly improves both overall and recurrence free survival. While systemic chemotherapy is generally considered ineffective for LAMN, little literature is available to support this notion. We evaluated outcomes for individuals with LAMN who did and did not receive systemic chemotherapy in combination with CRS+HIPEC.

**Methods:**

A multicenter retrospective cohort study was performed using the US HIPEC Collaborative that included patients with LAMN who underwent CRS+HIPEC. The overall survival (OS) and recurrence-free survival (RFS) of patients who did and did not receive systemic chemotherapy were compared. Survival and variables associated with survival were evaluated with the Kaplan-Meier analysis and cox regression, respectively.

**Results:**

Among the 529 included patients with LAMN, 63 (11.9%) received systemic chemotherapy and CRS+HIPEC, while 466 (88.1%) were treated with only CRS+HIPEC. Patients selected for systemic chemotherapy had a higher burden of disease (mean peritoneal cancer index: 18.8 +/- 8.6 versus 14.3 +/- 8.8, p<0.001). Patients who were not treated with chemotherapy had better mean OS and RFS (OS: 104.3 +/- 6.2 months, RFS: 84.9 +/- 6.6 months) compared to those who underwent systemic chemotherapy (OS: 70.2 +/- 6.8 months, RFS: 38 +/- 5.9 months, p<0.001). Increasing pre-operative CEA level (HR 1.012, p<0.001), higher completeness of cytoreduction score (reference CCR 0, CCR2 HR 34.175, p=0.001 and CCR3 HR 52.041, p=0.001), and treatment with systemic chemotherapy (HR 4.196, p=0.045) were associated with worse OS.

**Conclusions:**

In this multicenter retrospective study, the receipt of perioperative chemotherapy was associated with worse long-term outcomes among patients with LAMN undergoing CRS-HIPEC. Systemic chemotherapy may lead to patient deconditioning and contribute to worse long-term outcomes. It should not be recommended outside of a clinical trial.

## Introduction

Appendiceal mucinous neoplasms (AMNs) are rare tumors that can result in disseminated peritoneal disease, also known as pseudomyxoma peritonei (PMP). PMP is a clinical diagnosis, but the histologic nomenclature for AMNs is a source of inconsistency in the literature and clinical practice. This makes it difficult for providers to combine data and provide standardized, evidence-based treatment for patients. As such, there was a concerted effort by the Peritoneal Surface Oncology Group International (PSOGI) to reach a consensus for diagnostic terms for PMP secondary to AMNs (1).

The term “mucinous adenocarcinoma” is reserved for appendiceal lesions with infiltrative invasion, while LAMN (low grade appendiceal mucinous neoplasms) and HAMN (high grade appendiceal mucinous neoplasm) do not demonstrate invasion (1). Given the rarity of LAMN and HAMN, it can be difficult to diagnose outside of high-volume centers. Choudry et al. reviewed 115 AMNs from referring institutions at their own high-volume institution and found high discordance regarding correct terminology and tumor grade (2). On pathology review, 49% of patients referred with mucinous adenocarcinoma were downgraded to LAMN. Unfortunately, these inaccurate pathology assessments can be associated with over or undertreatment with systemic chemotherapy.

Cytoreductive surgery and hyperthermic intraperitoneal chemotherapy (CRS+HIPEC) is the standard of care in patients with PMP secondary to LAMN. However, systemic chemotherapy in treatment of LAMN PMP is unlikely to be efficacious due to the biologic, indolent nature of the disease (3–5). In fact, given the side effects and potentially absent therapeutic effect, patients who undergo systemic chemotherapy risk significant toxicity without any clear oncologic benefit. The result may translate to worse overall prognosis than those who only undergo CRS+HIPEC (6). Due to the rarity of this disease and decades of inconsistent nomenclature, data regarding LAMN is limited. Therefore, the objective of this study was to evaluate long term outcomes in patients with LAMN *with PMP* who received perioperative systemic chemotherapy and underwent CRS-HIPEC utilizing a large multi-institutional database.

## Methods

### Data source and patient selection

The United States HIPEC Collaborative database is a retrospective, multi-institutional database comprising data on patients who underwent CRS+HIPEC from 2000-2017 (7). There are 12 participating institutions that include: The Ohio State University, University of California San Diego, Mayo Clinic, MD Anderson Cancer Center, University of Cincinnati, Moffitt Cancer Center, Medical College of Wisconsin, Emory University, University of Massachusetts, Johns Hopkins University, City of Hope, and University of Wisconsin. Institutional review board approval was obtained from each institution.IRB permission was obtained to use this database for our study (IRB# 2017C0197). The US HIPEC Collaborative database was queried for patients aged ≥18 years old and diagnosed with LAMN who underwent CRS-HIPEC between 2000-2017.

### Patient variables

All patients in this database were treated with CRS+HIPEC. Patients with LAMN *with PMP* were divided into two cohorts based on whether or not they were treated with systemic perioperative chemotherapy (neoadjuvant and/or adjuvant). Patient clinicodemographic variables, tumor characteristics, treatment related data, and long-term outcomes were collected. Age, sex, race, health insurance, ECOG performance status, Charleson-Deyo score, pre-operative carcinoembryonic antigen (CEA) level, peritoneal cancer index (PCI) score at the time of CRS, post-operative complications, and completeness of cytoreduction (CCR) were included in the analysis. Long-term outcomes included overall survival (OS) and recurrence free survival (RFS). CCR can impact survival given that higher CCR scores indicate residual disease at the end of cytoreductive surgery. As such, a sub-analysis comparing OS of patients with low (CCR 0 or 1) and high (CCR 2 or 3) scores stratified by the receipt of chemotherapy was included in the analysis.

### Statistics

Categorical and continuous variables between patients who were treated with or without systemic chemotherapy were compared using the chi-square and independent samples t-test, respectively. Kaplan-Meier curves were created for OS and RFS. Cox regression univariable and multivariable analysis (proportional hazard model) was utilized to evaluate factors associated with worse overall survival. All statistics were performed with SPSS Version 29.0.1.0 (IBM).

## Results

### LAMN with PMP population demographics

Among 529 patients with LAMN who underwent CRS-HIPEC, 466 (88.1%) did not receive systemic chemotherapy (NSC), whereas 63 (11.9%) received systemic chemotherapy (SC, **Table 1**). Complete clinical, demographic, and pathologic characteristics are listed in **Table 1**. The NSC cohort had an average age of 55.5 +/- 12.4 years, was primarily female (n=290, 62.2%), Caucasian (n=395, 84.8), and had private health insurance (n=300, 64.4%). The SC cohort had an average age of 55.7 +/- 11.3 years, were primarily Caucasian (n=48, 76.2%), with private health insurance (n=44, 69.8%), and about half were female (n=31, 49.2%). Of the demographic data, there was a difference between the NSC and SC cohort regarding race. The SC cohort had a higher proportion of minority patients (23.8%) compared to the NSC cohort (15.2%, p<0.001). While there was no difference between the mean pre-operative CEA level between the two cohorts (NSC: 20.9 +/- 52.5 versus SC: 30.3 +/- 59.1, p=0.278), the SC cohort had a higher mean PCI (18.8 +/- 8.6) compared to the NSC cohort (14.3 +/- 8.8, p<0.001).

**Table 1:** Clinicopathologic data comparing patients with low grade appendiceal mucinous neoplasms (LAMN) with pseudomyxoma peritonei who were treated with CRS+HIPEC alone versus CRS+HIPEC with systemic chemotherapy.

	No Systemic Chemotherapy (n=466, %)	Systemic Chemotherapy (n=63, %)	p value
**Age (Mean +/- SD, years)**	55.5 +/- 12.4	55.7 +/- 11.3	0.920
**Pre-operative CEA Level (Mean +/- SD)**	20.9 +/- 52.5	30.3 +/- 59.1	0.278
**PCI (Mean +/- SD)**	14.3 +/- 8.8	18.8 +/- 8.6	<0.001
**Sex**			0.047
Male	176 (37.8)	32 (50.8)	
Female	290 (62.2)	31 (49.2)	
**Race**			<0.001
Caucasian	395 (84.8)	48 (76.2)	
African American	13 (2.8)	9 (14.3)	
Asian	22 (4.7)	4 (6.3)	
Hispanic	19 (4.1)	0 (0)	
Other	13 (2.8)	2 (3.2)	
Unknown	4 (0.9)	0 (0)	
**Health Insurance**			0.743
Private Insurance	300 (64.4)	44 (69.8)	
Government Insurance	127 (27.3)	16 (25.4)	
Uninsured	14 (3)	1 (1.6)	
Unknown	25 (5.4)	2 (3.2)	
**ECOG Performance Status**			0.119
0	243 (52.1)	33 (52.4)	
1	101 (21.7)	20 (31.7)	
2	12 (2.6)	3 (4.8)	
3	3 (0.6)	1 (1.6)	
4	1 (0.2)	0 (0)	
Unknown	106 (22.7)	6 (9.5)	
**Charleson-Deyo Score**			0.153
0	130 (27.9)	11 (17.5)	
1	121 (26)	22 (34.9)	
2	131 (28.1)	15 (23.8)	
3+	84 (18)	15 (23.8)	
**Timing of Chemotherapy**			N/A
Adjuvant	–	21 (33.3)	
Neoadjuvant	–	34 (53.9)	
Perioperative (neoadjuvant and adjuvant)	–	8 (12.6)	
**Completeness of Cytoreduction**			<0.001
0	279 (59.9)	23 (36.5)	
1	115 (24.7)	19 (30.2)	
2	23 (4.9)	7 (11.1)	
3	14 (3)	7 (11.1)	
Unknown	35 (7.5)	7 (11.1)	

### Overall survival and recurrence free survival of the LAMN with PMP population

**Figure 1:**
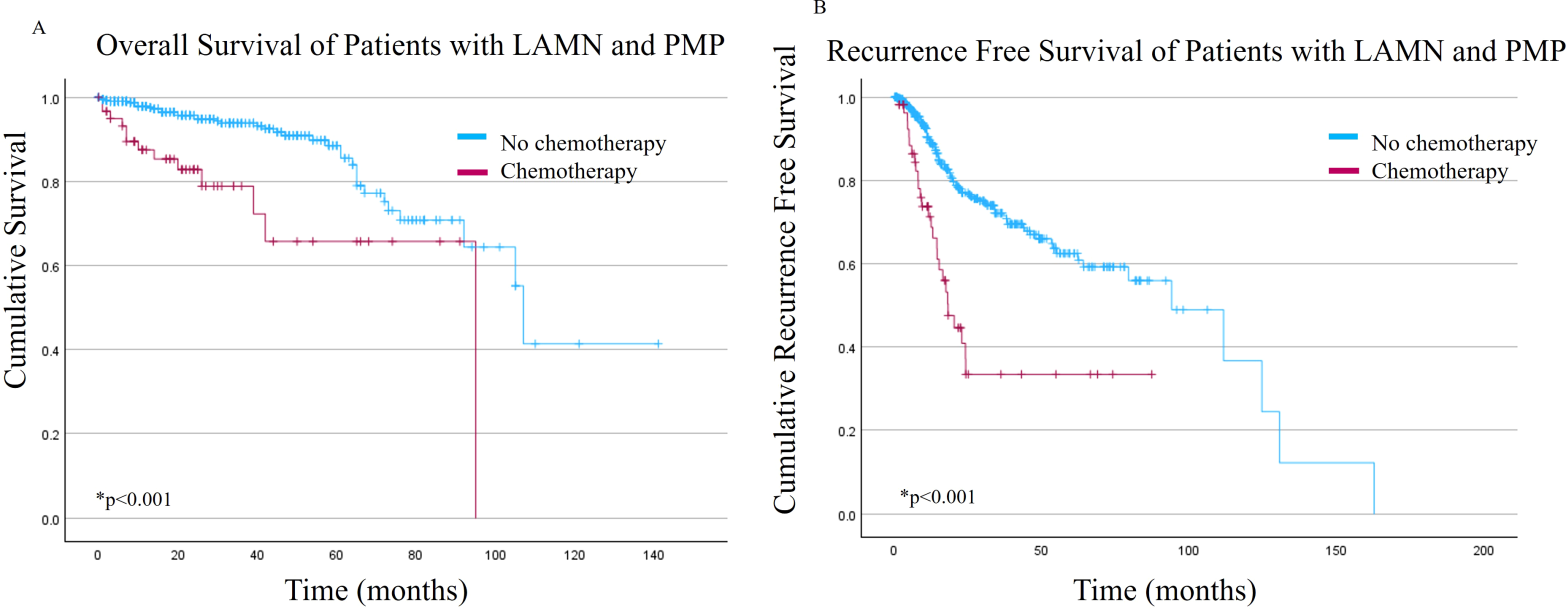
**(A)** Overall survival and **(B)** Recurrence free survival of patients with low grade appendiceal mucinous neoplasms (LAMN) with pseudomyxoma peritonei (PMP) stratified by receipt of systemic chemotherapy.

The NSC cohort had better OS and RFS compared to the SC cohort (**Figures 1A, B**, respectively). The mean OS was 104.3 +/- 6.2 months versus 70.2 +/- 6.8 months for NSC and SC, respectively (p<0.001). Mean RFS was 84.9 +/- 6.6 months versus 38 +/- 5.9 months for the NSC and SC, respectively (p<0.001).

### Cox regression analysis for the LAMN with PMP population

**Table 2** reports factors associated with OS based on Cox regression analysis. On univariable analysis, age, health insurance, Charleson-Deyo score, and post-operative complications were not associated with survival outcomes. Pre-operative CEA level, PCI, sex, race, ECOG performance status, completeness of cytoreduction (CCR), and treatment with systemic chemotherapy were all significant on the univariable analysis and therefore included on the multivariable analysis. On multivariable Cox regression, increasing pre-operative CEA (HR 1.012. CI 1.005 – 1.019, p<0.001), ECOG score of 1 (reference: 0, HR 5.023, CI 1.375 – 18.347, p=0.015), higher CCR score (reference: CCR 0; CCR 2 HR 34.175, CI 3.952 – 295.504, p=0.001, CCR 3 HR 52.041, CI 4.653 – 582.013, p=0.001), and treatment with systemic chemotherapy (HR 4.196, CI 1.033 – 17.048, p=0.045) were associated with worse overall survival.

**Table 2:** Univariable and multivariable cox regression evaluating variables associated with overall survival in patients with low grade appendiceal mucinous neoplasms (LAMN) with pseudomyxoma peritonei.

	Univariable Analysis			Multivariable Analysis		
	Hazard Ratio	Confidence Interval	p value	Hazard Ratio	Confidence Interval	p value
**Age (Mean +/- SD, years)**	1.026	0.999 – 1.053	0.058			
**Pre-operative CEA Level (Mean +/- SD)**	1.011	1.007 – 1.015	<0.001	**1.012**	**1.005 – 1.019**	**<0.001**
**PCI (Mean +/- SD)**	1.060	1.022 – 1.098	0.002	0.937	0.854 -.1.029	0.172
Sex						
Female	Reference			Reference		
Male	1.823	1.041 – 3.190	0.036	3.133	0.901 – 10.900	0.073
Race						
Caucasian	Reference			Reference		
African American	3.123	1.109 – 8.792	0.031	7.185	0.993 – 52.007	0.051
Asian	1.242	0.379 – 4.077	0.720	0.178	0.008 – 3.795	0.269
Hispanic	*	*	*	*	*	*
Other	*	*	*	*	*	*
Unknown	*	*	*	*	*	*
Health Insurance						
Private Insurance	Reference					
Government Insurance	1.096	0.609 – 1.974	0.759			
Uninsured	0.508	0.069 – 3.734	0.506			
Unknown	*	*	*			
ECOG Performance Status						
0	Reference			Reference		
1	1.864	0.983 – 3.534	0.056	**5.023**	**1.375 – 18.347**	**0.015**
2	3.023	1.018 – 8.975	0.046	2.209	0.193 – 25.281	0.524
3	10.941	1.437 – 83.276	0.021	*	*	*
4	*	*	*	*	*	*
Unknown	0.422	0.157 – 1.130	0.086	1.568	0.218 – 11.270	0.655
Charleson-Deyo Score						
0	Reference					
1	0.578	0.254 – 1.316	0.191			
2	0.717	0.319 – 1.608	0.419			
3+	1.624	0.764 – 3.451	0.208			
Post-operative Complications						
No	Reference					
Yes	1.092	0.615 – 1.942	0.763			
Unknown	1.560	0.206 – 11.793	0.666			
Completeness of Cytoreduction						
0	Reference			Reference		
1	2.145	0.910 – 5.059	0.081	2.905	0.454 – 18.599	0.260
2	8.843	3.872 – 20.194	<0.001	**34.175**	**3.952 – 295.504**	**0.001**
3	20.512	8.960 – 46.958	<0.001	**52.041**	**4.653 – 582.013**	**0.001**
Unknown	5.929	2.042 – 17.212	0.001	**41.110**	**2.680 – 630.571**	**0.008**
Systemic Chemotherapy						
No	Reference			Reference		
Yes	3.139	1.657 – 5.947	<0.001	**4.196**	**1.033 – 17.048**	**0.045**

Bold values indicate statistical significance on multivariable analysis.

### Overall survival of the LAMN with PMP population with CCR 2 or 3

**Figure 2:**
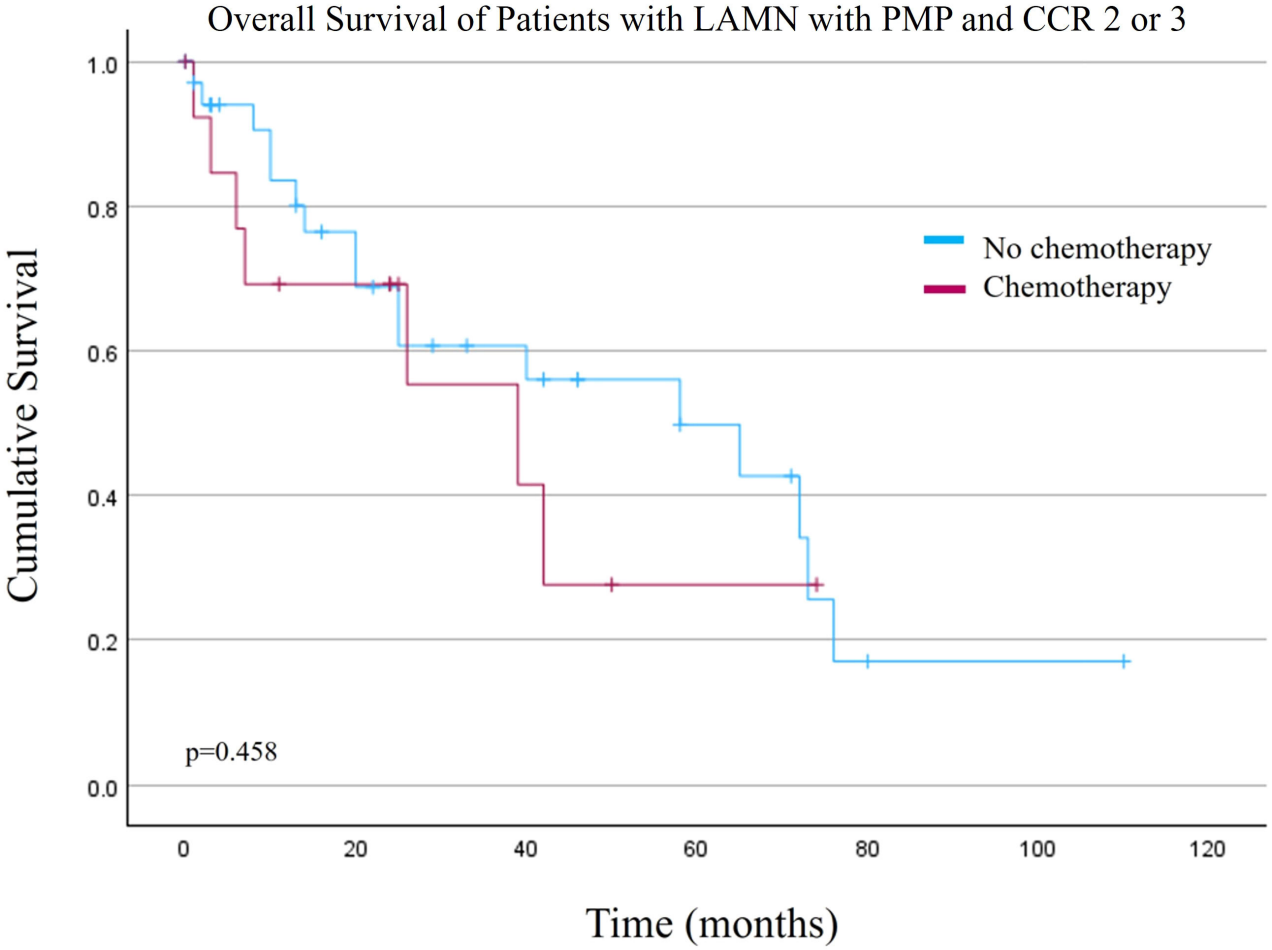
Overall survival of patients with low grade appendiceal mucinous neoplasms (LAMN) with pseudomyxoma peritonei (PMP) and macroscopic disease after cytoreduction surgery and hyperthermic intraperitoneal chemotherapy (Completeness of Cytoreduction (CCR) 2 or 3).

Based on the Cox regression results, an additional survival analysis was performed for patients with CCR 2 or 3 (n=51). There was no difference in overall survival for this cohort of patients when comparing the NSC versus SC cohorts (**Figure 2**, p=0.458). Mean OS was 53.3 +/- 7.5 months versus 36.6 +/- 8.5 months for the NSC and SC cohorts, respectively.

## Discussion

CRS+HIPEC is the standard of care for patients with PMP secondary to LAMN. The use of systemic chemotherapy for this disease process is controversial since LAMN with PMP is non-invasive by definition and typically has an indolent clinical course (1). Nevertheless, perhaps because of confusion based on nomenclature, its rarity, or the impetus to provide aggressive treatment for an advanced peritoneal surface malignancy, patients with LAMN and PMP still receive systemic chemotherapy even at academic medical centers. Acknowledging the limitations of a retrospective cohort study in which patients are not selected for treatment randomly, the findings in this current study suggest that perioperative systemic chemotherapy should not be used for patients with LAMN and pseudomyxoma peritonei undergoing CRS-HIPEC.

The PSOGI consensus terminology advocates for a classification system of mucinous appendiceal neoplasms based on histologic features, including infiltrative invasion, cytologic grade, tumor cellularity, angiolymphatic invasion, perineural invasion, and signet ring cells. One of the central components of this classification system is the distinction between “pushing” and “infiltrative” invasion. The term “adenocarcinoma” is exclusively used for patients with infiltrative invasion, while HAMN or LAMN refer to appendiceal lesions beyond the mucosa without infiltrative invasion (1). Pseudomyxoma peritonei (PMP) is a clinical diagnosis defined as mucinous ascites and peritoneal implants arising from mucinous appendiceal neoplasms (e.g. LAMN, HAMN) or mucinous adenocarcinomas. However, the terminology for peritoneal spread remains controversial and at times confusing. Based on PSOGI guidelines, there is a four-tiered system to pathologically describe PMP. The term “low-grade mucinous carcinoma peritonei” is recommended to describe low-grade appendiceal lesions with peritoneal spread (1). While “carcinoma” is colloquially associated with an invasive appendiceal cancer, here it simply refers to disseminated peritoneal disease secondary to LAMN. This unclear phrasing may result in overtreatment with systemic chemotherapy. Patients are subjected to the risks and deconditioning associated with chemotherapy without deriving an oncologic benefit. This may be one reason why 12% of patients in our database received systemic chemotherapy. Other reasons may include changes in guideline-driven care over time or attempts to downstage patients with poor prognostic indicators despite lack of malignant indication, as evidenced by the higher mean PCI in patients who underwent chemotherapy.

In addition to systemic chemotherapy, elevated pre-operative CEA level and higher post-operative CCR score were associated with worse overall survival. This is congruent with existing evidence (8–10). It is unclear though if LAMN, HAMN, and mucinous appendiceal adenocarcinoma are phases of the same disease or separate biologic processes. Recent studies explore the genetic landscape of appendiceal neoplasms and subsets defined by their mutational status may confer worse overall survival (11, 12). If this represents a stepwise progression, then perhaps residual disease (higher CCR score) is an opportunity for a critical mutational event resulting in appendiceal adenocarcinoma. However, in our database survival for patients with residual macroscopic disease after CRS+/-HIPEC (CCR 2 or 3) showed no difference in OS between patients who did or did not receive systemic chemotherapy. As such, further research to better classify the subtypes of AMNs and appendiceal adenocarcinomas may be able to predict recurrence patterns and guide treatment decisions for repeat CRS+HIPEC.

This study offers valuable insight into the treatment of patients with LAMN, but it is important to acknowledge the limitations due to its retrospective design. This database was created by 12 institutions, therefore there are some missing data and miscoded information. Due to this, the data should be interpreted with caution since missing variables have the potential to impact our analysis. Furthermore, recurrence data can be difficult to capture in a retrospective database. This is one of the reasons overall survival was included in the analysis. It is not well understood if LAMN or HAMN have the potential to develop into appendiceal adenocarcinoma and it is possible that patients with LAMN and PMP at initial CRS/HIPEC progressed to appendiceal adenocarcinoma and required systemic chemotherapy. This type of data is difficult to capture in a retrospective database. Second, this database includes patients from a large time period (2000-2017). As such, treatment decisions may be influenced by evolving terminology and consensus guidelines. This database also does not capture patients from low-volume institutions where the rate of over treatment with systemic chemotherapy for LAMN and PMP may be higher. Finally, there were no genetic data available for these patients, likely because it is not routine to send LAMN samples out for genetic testing.

There is little to no data in the literature on the treatment of patients with LAMN, and what does exist is focused on small, single-center studies. This study shows that patients with LAMN and PMP who receive systemic chemotherapy and CRS+HIPEC have worse overall and recurrence free survival compared to those treated with CRS+HIPEC alone. This is conceivably because patients take on the risk of toxic chemotherapy without deriving an oncologic benefit. In addition to validating this work with larger cohorts, prospective data with more nuanced variables would may be able to identify variables associated with the receipt of chemotherapy in this patient population. Furthermore, studies show discordance between low-volume and high-volume centers regarding pathology interpretation of appendiceal mucinous neoplasms (2, 13, 14). Despite attempts to establish consensus diagnoses, there still remains controversy and confusion regarding terminology. Overinterpretation of LAMN as appendiceal adenocarcinoma or incorrectly interpreting “low-grade mucinous carcinoma peritonei” as an invasive process results in unnecessary systemic chemotherapy in patients who will derive no oncologic benefit. Patients with AMNs or appendiceal adenocarcinomas should be referred to high-volume centers where pathology can be reviewed and care guided by a multi-disciplinary team.

## Data Availability

The original contributions presented in the study are included in the article/supplementary material. Further inquiries can be directed to the corresponding author/s.
